# Double PitNETs: A Case Report and Literature Review

**DOI:** 10.3390/cancers17040675

**Published:** 2025-02-17

**Authors:** Mitsuru Nishiyama, Noriaki Fukuhara, Hiroshi Nishioka, Shozo Yamada

**Affiliations:** 1 Department of Endocrinology, Metabolism and Nephrology, Kochi Medical School, Kochi University, 1-185, Kohasu, Oko-cho, Nankoku City 783-8505, Kochi, Japan; 2 Health Care Center, Kochi University, 1-5-2, Akebono-cho, Kochi City 780-8520, Kochi, Japan; 3 Department of Hypothalamic and Pituitary Surgery, Toranomon Hospital, Minato-ku, Tokyo 105-8470, Japan; 4 Neurosurgery Center, Moriyama Memorial Hospital, Edogawa-ku, Tokyo 134-0081, Japan

**Keywords:** double pituitary neuroendocrine tumor (PitNET), double pituitary adenoma, acromegaly, Cushing’s disease, prolactinoma, TSH-producing tumor, non-functioning pituitary tumor

## Abstract

Double pituitary neuroendocrine tumors (double PitNETs) are two distinct tumors in the same gland and are infrequent in clinical practice. In typical double PitNETs, an MRI detects two separate tumors that are diagnosed by pathology; they could also appear as a single tumor, and pathology would then identify the two independent tumors. A literature review was conducted, and 142 cases were analyzed to determine the characteristics of double PitNETs. Of these cases, acromegaly (45.5%) was the most common clinical feature, followed by Cushing’s disease (35.1%) and prolactinoma (17.9%), indicating that double PitNETs are usually noticed by hormonal excess symptoms due to at least one functional tumor. The pathological analysis of 284 tumors showed that somatotroph (28.9%) and corticotroph (26.8%) tumors were predominant, with a recent increase in the proportion of gonadotroph tumors. Regarding transcription factors, 51.1% were of GH-PRL-TSH PIT1-lineage, 26.1% ACTH TPIT-lineage, and 17.9% LH-FSH SF1-lineage. The radiological analysis of 82 cases revealed that double tumors (45.1%) and single tumors (47.6%) were comparable, suggesting that double PitNETs are often detected as a single tumor, and attention should be paid to hidden micro-tumors during surgery. Double PitNETs are complicated by a wide variety of clinical, radiological, and pathological findings, but diagnostic and therapeutic approaches are advancing.

## Simple Summary

Pituitary lesions include tumors, cysts, and inflammation, with a very low incidence of double pituitary neuroendocrine tumors (double PitNETs). Double PitNETs are a challenging condition in clinical practice due to their wide variety of clinical, pathological, and radiological features. We performed a literature review and identified 142 cases of double PitNETs, including the present case, and analyzed the clinical manifestations, pathological findings of each tumor, and radiological features of cases using MRI findings. Despite the infrequency of clinically evident double PitNETs, the number of reported cases is increasing, and the present analyses indicate a recent increase in the proportion of endocrine-inactive cases and gonadotroph tumors. This complicated disease is becoming clearer, and endocrinologists, pituitary neurosurgeons, and pathologists need to understand the unique features of double PitNETs.

## 1. Introduction

Pituitary neuroendocrine tumors (PitNETs) are found in 10% of autopsy cases, are clinically evident in 0.1% of the population, and are classified as either functioning tumors based on the hormone-produced (GH, PRL, TSH, ACTH, or LH/FSH) or non-functioning tumors, most of which have a benign course [1,2]. PitNETs are usually diagnosed by specific features due to hormonal excess or local symptoms (e.g., headache and visual disturbances) caused by tumor compression. Prolactinomas and non-functioning tumors present with the most common clinical manifestations, followed by acromegaly, and Cushing’s disease. Regarding the pathological diagnosis, PitNETs can be largely divided into the following three groups based on pituitary transcription factor positivity: PIT1-lineage (somatotroph, lactotroph, and thyrotroph tumors), TPIT-lineage (corticotroph tumors), and SF1-lineage (gonadotroph tumors), according to the 2022 WHO classification [3,4].

Double PitNETs comprise two distinct tumors in the same gland. While the term “double pituitary adenoma” is commonly used in the literature, the 2022 WHO classification suggests the use of “multiple synchronous PitNET/adenomas of distinct lineages” [3]. It is a challenging condition for clinicians, and an appropriate clinical diagnosis of hormonal abnormality is essential, along with pathological evaluation. In typical double PitNETs, magnetic resonance imaging (MRI) detects two separate tumors that are then diagnosed by pathology; however, these lesions can appear as a single tumor on the MRI, but pathology identifies two independent tumors [5,6]. Transcription factors, along with hormonal staining, are also useful in the diagnosis of double PitNETs, and tumors that are adjacent on the MRI can be clearly diagnosed as double PitNETs, especially when there is a combination of different lineages [7]. However, if both adjacent tumors are positive for the same lineage of transcription factors, this is not a useful basis for distinguishing double PitNETs from the heterogeneity of a single PitNET.

The incidence of clinically evident double PitNETs is extremely low. With the recent developments in pituitary medical techniques, the number of reported cases of double PitNETs has increased [5,6]; however, their clinical, radiological, and pathological features have not been fully characterized. In addition, pituitary transcription factors have received attention in PitNET diagnosis following the revision of the 2022 WHO classification of PitNET [3,7] but have not been well studied in double PitNETs. In this review, we present a typical case of double PitNETs, perform a literature review, and discuss this complicated condition.

## 2. Case Presentation

**Figure 1:**
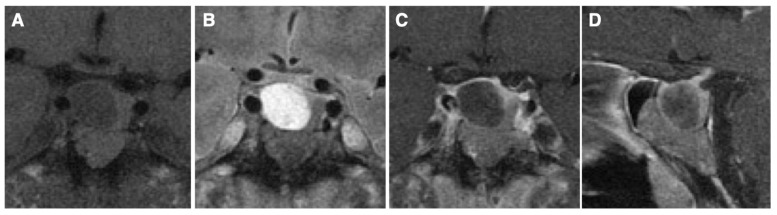
Magnetic resonance images: T1-weighted (**A**), T2-weighted (**B**), gadolinium-enhanced T1-weighted (**C**) in the coronal section, and T1-weighted image in the sagittal section (**D**), showing a 2.5 cm diameter macro-tumor with suspected cystic degeneration in the upper portion.

**Figure 2:**
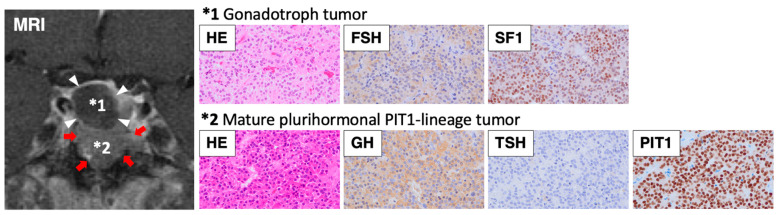
Microscopic examination of the pituitary tumor with hematoxylin/eosin (HE) staining and immunohistochemical staining of FSH, SF1, GH, TSH, and PIT1 (magnification ×400). The upper lesion (*1) in the MRI (same as Figure 1) was determined as a gonadotroph tumor (indicated by white arrowheads in the MRI; FSH+, SF1+ in pathology), and the lower lesion (*2) was determined as a mature plurihormonal PIT1-lineage tumor (indicated by red arrows in the MRI; GH++, TSH±, PIT1+ in pathology).

A 51-year-old man presented with clinical features typical of acromegaly. Serum GH and IGF-1 levels were markedly elevated (GH 81.3 ng/mL [reference range < 2.47]; IGF-1 1230 ng/mL [87–243], SD score 11.1), and GH was not suppressed during the oral glucose tolerance test. Additionally, the thyroid hormone levels were elevated (FT3 5.69 pg/mL [2.3–4.3]; FT4 2.79 ng/dL [0.9–1.7]) with the inappropriate secretion of TSH (TSH 1.79 μIU/mL [0.61–4.23]), and the TSH receptor antibodies were negative. Circulating ACTH, cortisol, PRL, LH, FSH, and testosterone levels were within the normal ranges. MRI revealed a 2.5 cm diameter pituitary lesion with suprasellar extension, suggesting a pituitary tumor with cystic degeneration (Figure 1). No visual field impairment was observed. It was diagnosed as a functional pituitary tumor secreting both GH and TSH, and the preoperative administration of a somatostatin analogue (200 µg/day) resulted in the normalization of thyroid function. The patient underwent transsphenoidal surgery, which revealed double PitNETs—one in the lower portion, and the other in the upper portion of the pituitary, which was considered a cyst before surgery. Pathological examination confirmed double PitNETs comprising a GH/TSH secreting mature plurihormonal PIT1-lineage tumor (GH++, TSH±, PRL-, PIT1+) and a non-functioning gonadotroph tumor (FSH+, SF1+) (Figure 2). An MRI showed no residual tumor postoperatively, and the hyperthyroidism was normalized, but the GH and IGF-I levels were not completely normalized (GH 1.71 ng/mL; IGF-I 291 ng/mL, SD score 2.8); therefore, a long-acting somatostatin analogue (10 mg/month) was initiated, following which GH and IGF-1 were well controlled.

## 3. Literature Review

A systematic search of the historical literature was conducted in PubMed, the United States National Library of Medicine, using “double/multiple pituitary adenomas” and “double/multiple pituitary neuroendocrine tumors (PitNETs)”, limited to 1980 and later. Clinical case reports and case series of histologically proven double/multiple PitNETs were analyzed. Cases with radiological evidence of double pituitary lesions but no pathological findings were excluded. Autopsy cases were not analyzed but are discussed in the epidemiology section.

We found 144 cases of multiple PitNETs, with 141 double [8,9,10,11,12,13,14,15,16,17,18,19,20,21,22,23,24,25,26,27,28,29,30,31,32,33,34,35,36,37,38,39,40,41,42,43,44,45,46,47,48,49,50,51,52,53] and 3 triple PitNETs [39,54,55] in 48 studies. We analyzed 142 cases of double PitNETs, including the present case, and excluded triple PitNETs from further analysis. In double PitNETs, most cases showed two lesions at the time of diagnosis (synchronous); however, in six cases, the second tumor occurred at different times (metachronous) [32,38,42,44,49,53]. While 58 double PitNETs cases were reported between 1980 and 2009 (30 years), 84 were reported between 2010 and 2024 (15 years). Recent advances in both the radiological and pathological technologies for pituitary tumors may have increased the number of newly diagnosed cases of double PitNETs [3,23,38].

The clinical features of double PitNETs were analyzed in 134 patients with documented clinical symptoms and endocrine examinations. To clarify the pathological features, a combination of positive immunohistochemical (IHC) staining for each pituitary hormone was analyzed in 284 tumors (142 cases). Recent reports have recognized the importance of transcription factors [1,2,3,4,7], and the combinations of double PitNETs classified by cell lineage were also analyzed. Since transcription factors were examined only in 42 cases (29.6%), we interpreted GH-PRL-TSH positivity as PIT1 lineage, ACTH positivity as TPIT lineage, and LH-FSH positivity as SF1 lineage. In addition, the imaging findings of double PitNETs were analyzed in 82 cases examined by MRI, and cases based on CT evaluation and cases with metachronous onset were excluded. Macro-tumors were defined as those at least 10 mm in diameter and micro-tumors as those less than 10 mm in diameter.

## 4. Statistical Analysis

Continuous variables were expressed as mean ± standard deviation (SD), and categorical variables were expressed as numbers and percentages. Clinical, pathological, and radiological characteristics in double PitNETs were compared between older (1980–2009) and recent (2010–2024) years, and the statistical analyses in a 2 × 2 table were assessed using the chi-squared test, with *p* value < 0.05 considered statistically significant. All calculations were performed using JMP version 14.0 software (SAS institute, Cary, NC, USA).

## 5. Epidemiology

Recent epidemiological analyses have revealed that a single PitNET is found in 10% of autopsy cases, but most lesions are less than 3 mm and macro-tumors are rare. On the other hand, PitNETs are clinically evident with an incidence of 0.1% in the community, and the number of PitNETs has increased in recent years owing to advances in the medical technology for diagnosing pituitary tumors [1,2].

The prevalence of multiple, incidentally detected PitNETs in autopsy samples varies widely. Burrow et al. analyzed 120 autopsy cases and found eight multiple PitNETs (6.7%), with serial 1 mm sections [56]. Kontogeorgos et al. analyzed 470 randomly autopsied pituitary glands and found a 0.9% prevalence of multiple PitNETs, most of which were less than 3 mm in size [57]. More recently, Buurman and Saeger found 316 PitNETs, including 17 multiple PitNETs (0.55%), in 3048 autopsy cases [58].

Since the incidence of multiple clinically evident PitNETs is very low, their prevalence is usually expressed as a percentage of the total number of PitNETs analyzed in surgically resected cases. Several studies have reported on the frequency of multiple PitNETs, and the analyses prior to 2010 have shown their incidence ranging from 0.16 to 2.6% [5,8,11,15,17,21,27,29]. Mete et al. analyzed pathology records from 2001 to 2016 retrospectively and found 13 multiple PitNETs (1.2%) in 1055 cases [39], while Zieliński et al. found 22 double PitNETs (0.67%) in 3270 cases [42]. Schöning et al. examined multiple PitNETs and found the prevalence was 0.58% in 3610 autopsy cases and 0.13% in 12,673 surgical cases [59].

To summarize the recent analyses, multiple PitNETs are found in approximately 0.6–0.9% of autopsy cases and 0.13–1.2% of surgically resected PitNETs; however, because non-functioning micro-tumors are rarely operated on, their prevalence may be higher than reported.

## 6. Pathogenesis

Although most single PitNETs are sporadic, their molecular pathogenesis has not been clarified. Activating somatic mutations are commonly observed in somatotroph tumors in the guanine nucleotide activating subunit (*GNAS*) and corticotroph tumors in ubiquitin-specific protease 8 (*USP8*) [60,61,62]. Germline mutations cause syndromic pituitary tumors including MEN1 (multiple endocrine neoplasia type 1) (*MEN1*), MEN4 (cyclin-dependent kinase inhibitor 1B: *CDKN1B*), Carney complex (type 1 alpha regulatory subunit of protein kinase A: *PRKAR1A*), McCune–Albright syndrome (*GNAS*), and familial isolated pituitary adenoma (FIPA) (aryl hydrocarbon receptor-interacting protein: *AIP*) [1,60]. In our literature review, the majority of reported double PitNETs were sporadic, with no described somatic or germline mutations, and only seven cases had germline mutations (MEN1: 5, loss of heterozygosity on chromosome 11q13: 1, Carney complex: 1) [13,14,15,17,21,39,42]. MEN1 may be more frequent in double PitNETs than expected in adult patients [5,63], suggesting that cases with *MEN1* mutation should be aggressively examined.

The mechanism of double PitNETs development has been discussed in previous reports, and the following three hypotheses have been proposed: (1) multi-hit theory (a multicentric occurrence in the same pituitary gland), (2) trans-differentiation theory (a different clonal proliferation originating from one PitNET), and (3) induction theory (one PitNET induces the formation of another) [5,64]. Single PitNETs expressing transcription factors of multiple lineages are extremely rare [65,66,67], and it is more common to have double PitNETs, suggesting a low probability of trans-differentiation theory [3]. GH and IGF-1 can induce tumor development by promoting cell proliferation [68], and multiple PitNETs are relatively frequent (1.6–3.3%) in Cushing’s disease [17], suggesting the possibility of GH or ACTH related induction theory although supportive evidence is lacking. Hagel et al. analyzed the genetic background of two tumors in the same gland in four cases of double PitNETs and found that copy numbers and global methylation patterns were completely different [45]. These results suggest that double PitNETs may originate from distinct cell types, which supports the idea of the multi-hit theory.

The pathogenesis of double PitNETs is unknown, but the high prevalence of PitNETs in autopsy cases indicates the possibility of a multi-hit theory. Since supportive evidence is scarce for trans-differentiation and induction theories, it is more likely that the two tumors simply occur in the same gland.

## 7. Clinical Phenotype

Among the double PitNETs cases, the mean ages were 47.3 ± 15.4 years old (ranges: from 13 to 81 years old), and there was a female predominance (60 males, 79 females, and 3 unknown). The same trend was observed when the older and recent years were separated: mean ages were 43.1 ± 15.6 years old, 22 males and 33 females in 1980–2009; and 47.3 ± 15.4 years old, 38 males and 46 females in 2010–2024.

In the current literature review, acromegaly (45.5%) and Cushing’s disease (35.1%) were the most frequent clinical features of double PitNETs, followed by prolactinoma (17.9%), endocrine-inactive tumors (12.7%), and TSHoma (1.5%) (Table 1). As in the previous reviews, nearly 80% of all cases showed clinical symptoms associated with GH or ACTH excess [5,32,52,64], suggesting that these features are often triggers for the diagnosis of double PitNETs. These results are inconsistent with epidemiological findings that prolactinomas and non-functioning tumors are common in single PitNETs. The fact that prolactinomas are treated with medications and not confirmed by surgery and that non-functioning micro-tumors are less likely to be detected during surgery may be the reason for their low frequency.

There are several unique combinations of clinical symptoms in patients with double PitNETs (Table 1). Three cases of combined acromegaly and Cushing’s disease have been reported, two of which were acromegaly, and one was Cushing’s dominant [10,31,41]. A clinical combination of acromegaly and Cushing’s disease suggests double PitNETs because of their different lineages. Six cases with a combination of acromegaly and prolactinoma were evident [11,15,28,29], and the possibility of double PitNETs with somatotroph and lactotroph tumors should be considered, although somatotroph tumors producing GH and PRL simultaneously are common [1,2]. Seven cases with a combination of Cushing’s disease and prolactinoma have been described [11,12,16,17,32,38,45].

Most double PitNETs show clinical features related to hormonal excess, but there are a certain number of cases with endocrine-inactive tumors (12.7%). These cases are recognized by local symptoms due to the tumors or detected as incidental tumors in imaging studies [11,17,26,27,38,39,42,47]. Thyrotoxic symptoms in double PitNETs are rare (1.5%) [17,42], probably due to the low incidence of thyrotroph tumors in PitNETs [1,2]. In our present case, the marked elevation of GH/IGF-1 and the mild elevation of the thyroid hormones due to GH/TSH produced a PitNET-induced acromegaly phenotype without thyrotoxicosis. Additionally, only one case of gonadotropin excess has been reported [51].

A chronological comparison of clinical features in double PitNETs yielded the following results: acromegaly, 23 cases (39.7%); Cushing’s disease, 21 cases (35.2%); prolactinoma, 12 cases (20.7%); TSHoma, 1 case (1.7%); endocrine-inactive tumor, 3 cases (5.2%) in 1980–2009; and acromegaly, 38 cases (45.2%); Cushing’s disease, 26 cases (31.0%); prolactinoma, 12 cases (14.3%); TSHoma, 1 case (1.2%); endocrine-inactive tumor, 14 cases (16.7%) in 2010–2024. Of these results, the percentage of endocrine-inactive cases increased significantly in recent years compared to the older years (*p* = 0.04), while the other groups had the same proportion in both periods.

## 8. Pathological Findings

In 284 PitNETs (142 cases), combinations of positive immunohistochemical (IHC) staining for each pituitary hormone were analyzed (Table 2). The percentage of positive staining for each hormone was as follows: 82 GH-positive tumors (28.9%), 76 ACTH-positive tumors (26.8%), 50 PRL-positive tumors (17.6%), and 47 LH/FSH-positive tumors (16.5%), which were similar to previous reports [5,32,52]. These results are consistent with the high prevalence of acromegaly, Cushing’s disease, and prolactinoma in the clinical phenotype.

Case-based IHC results showed that 71 cases (50.0%) had any GH, 71 (50.0%) had any ACTH, 48 (33.8%) had any PRL, and 45 (31.7%) had any LH/FSH. Among these cases, the combinations of GH + ACTH (22 cases, 15.5%), GH + LH/FSH (22 cases, 15.5%), and ACTH + PRL (27 cases, 19.0%) were common, followed by GH + GH (11 cases, 7.7%), ACTH + LH/FSH (11 cases, 7.7%), and PRL + LH/FSH (8 cases, 5.6%). Although combinations of GH + ACTH and ACTH + PRL were relatively frequent, only a few cases presented with both the clinical features of these combinations (Table 1). The incidence of combined GH + PRL was relatively low (seven cases, 4.9%), whereas somatotroph tumors with the simultaneous production of GH and PRL are common [1,2]. Overall, somatotroph tumors are combined with a variety of tumors, but corticotroph tumors seem to be more specifically combined with lactotroph tumors. Although a link between estrogen excess and prolactinoma development has been noted [69], there is no evidence of lactotroph tumor induction by ACTH or cortisol excess. In the present case, somatotroph and gonadotroph tumors were observed, which is a common combination of double PitNETs, but it is infrequent to contain a plurihormonal tumor.

The analysis of each hormone staining by IHC, divided by chronological age (1980–2009 and 2010–2024), showed the following results: 35 GH-positive tumors (30.2%), 32 ACTH-positive tumors (27.6%), 27 PRL-positive tumors (23.3%), and 8 LH/FSH-positive tumors (6.9%) in 1980–2009; and 47 GH-positive tumors (28.0%), 44 ACTH-positive tumors (26.2%), 23 PRL-positive tumors (13.7%), and 39 LH/FSH-positive tumors (23.2%) in 2010–2024. Statistical analyses revealed that the percentage of LH/FSH-positive tumors increased while PRL-positive tumors decreased in recent years compared to older years (Table 3). These results indicate that the proportion of gonadotroph tumors has increased in recent years. Although double PitNETs are often diagnosed based on hormone excess symptoms such as acromegaly and Cushing’s disease, there may be an increasing number of cases diagnosed during the testing process for non-functioning PitNETs. It is also possible that the recent advances in pathology have resulted in more cases being properly diagnosed as gonadotroph tumors [70].

The determination of each hormone and transcription factor positivity are useful for detailed pathological diagnosis in double PitNETs [39,59]. Because transcription factors were examined only in restricted cases (42 cases, 29.6% of reported cases), we interpreted GH-PRL-TSH positivity as PIT1 lineage, ACTH positivity as TPIT lineage, and LH-FSH positivity as SF1 lineage. From the viewpoint of cell lineage classification, predicted positive results for each transcription factors were 143 PIT1-lineage tumors (51.1%), 73 TPIT-lineage tumors (26.1%), and 50 SF1-lineage tumors (17.9%). The frequency of these transcription factors was comparable to that reported in the previous studies [52,59], and the high proportion of GH and PRL staining may be reflected in the PIT1 positivity. Regarding a combination of transcription factors, PIT1 + TPIT (48 cases, 33.8%), PIT1 + SF1 (29 cases, 20.4%), and PIT1 + PIT1 (24 cases, 16.9%) were predominant. Analysis of transcription factors by chronological age also supported a recent trend toward an increasing proportion of SF1-positive gonadotroph tumors, as follows: PIT1 60.3%, TPIT 28.4%, and SF1 6.9% in 1980–2009; and PIT1 43.5%, TPIT 23.8%, SF1 25.0% in 2010–2024. Statistical analyses indicate that the percentage of SF1-lineage tumors increased significantly (*p* < 0.01), while PIT1-lineage tumors decreased (*p* < 0.01) in recent years compared to older years.

## 9. Radiological Findings

Pituitary imaging technology has advanced over the years, and MRI can be used to visualize lesions as small as a few millimeters. Contrast-enhanced MRI is used to identify pituitary micro-tumors, and the tumor area can be detected as a less-enhanced lesion. However, it can be difficult to diagnose pituitary tumors based on imaging findings alone, because pituitary lesions may include tumors, cysts, and inflammation. If imaging studies show two tumor-like lesions in the pituitary gland, they may be double PitNETs, but the presence of two different lesions cannot be excluded [59]. In the present case, a preoperative MRI suggested a pituitary tumor with cystic degeneration, but the intraoperative findings and pathology led to the diagnosis of double PitNETs (Figure 1 and Figure 2). Although the two tumors were in contact on the images, they differed in location and internal signal, and together with the pathological findings, this case was diagnosed as double PitNETs (distinct type). In contrast, a pathological examination may reveal double PitNETs (contiguous type) in patients thought to have a single tumor on preoperative MRI.

Imaging studies are useful in diagnosing double PitNETs and identifying the site of the lesion and are especially important in tumors causing Cushing’s disease. The treatment of Cushing’s disease is based on surgical resection, but the lesions are often small and may be difficult to identify even with an MRI, making it difficult to differentiate them from ectopic ACTH syndrome. Therefore, in cases where Cushing’s disease is suspected, evaluation with thin slice imaging using 3 Tesla (3T) MRI should be performed [71]. In addition, the usefulness of a thorough examination using spoiled gradient-recalled acquisition (SPGR) sequences, or methionine positron emission tomography (methionine-PET) fusion 3T-MRI has been reported [72,73]. It is problematic when either tumor is an ACTH-producing micro-tumor, and it can be difficult to detect the origin of Cushing’s disease on preoperative MRI [17,20,24,33,42,43]. In such cases, intraoperative attention should be paid to the presence of micro-tumors for possible double PitNETs. The IPSS helps determine whether ACTH excess is of pituitary origin [71].

The imaging findings of double PitNETs were analyzed in 82 cases that were examined using MRI (Table 4). The frequencies of cases observed as single tumors (contiguous type, 47.6%) and double tumors (distinct type, 45.1%) were similar, which is consistent with a previous report [42]. Unlike our analysis, Roberts et al. reported a predominance of the contiguous type in double PitNETs [38], probably because of differences in the analysis year. The chronological analysis of imaging findings showed no significant differences in the percentage of tumor type, as follows: contiguous, 13 cases (46.4%); distinct, 10 cases (35.7%) in 1980–2009; and contiguous, 26 cases (48.1%); distinct 27 cases (50.0%) in 1980–2009. One of the two tumors in the double PitNETs was a macro-tumor in 51 cases (62.2%), which was more frequent than cases with only micro-tumors (25 cases, 30.5%) in the present analysis. A previous review indicated that micro-tumors were more dominant than macro-tumors [5,52], and the difference between the present results and their reports may be explained by the case selection and year of analysis. Six cases involving Cushing’s disease were described, in which no tumor was detected on MRI, but double PitNETs were confirmed by pathological examination [17,42].

Collectively, it is relatively easy to consider double PitNETs when two separate lesions are observed on MRI, but they are often detected as a single macro- or micro-tumor. In the case of a single tumor, pathological examinations, including evaluation of hormones and transcription factors, are required to determine the presence of double PitNETs. It should also be noted that micro-tumors coexist with macro-tumors in some cases.

## 10. Treatment

The treatment for PitNETs is different for each disease and has progressed over the years, including surgery, medication, and radiation [2]. Surgery is the first choice of treatment for acromegaly, with medication (somatostatin analogues, dopamine agonists, GH receptor antagonists, etc.) and radiation for residual tumors or inoperable cases [74]. In the present case, surgery was effective, but mild elevations of GH and IGF-1 persisted, and a somatostatin analogue was added to restore the hormone excess. Surgery is the first-line therapy for Cushing’s disease and is essential for treatment algorithms. If a surgery fails to achieve remission, pasireotide, a somatostatin analogue, may be used as an ACTH inhibitor, but it frequently causes impaired glucose tolerance as a side effect. Cortisol synthase inhibitors have also been prescribed as an adrenal-targeted therapy [71]. Dopamine agonists are effective in the majority of prolactinoma cases, and surgical treatment is indicated for drug-resistant and giant prolactinomas [75,76]. The treatment of TSH-producing tumors involves surgery, and somatostatin analogues are also effective as drug therapies. Preoperative administration of somatostatin analogues is useful to normalize thyroid function [77], as in the present case. In the treatment of non-functioning PitNETs, surgical therapy is indicated in cases of concomitant visual field impairment or tumor enlargement, but an effective medication has not been established [2].

In double PitNETs, both pathologies need to be treated depending on the combination of tumors. In the present review, we included double PitNETs confirmed by pathology; therefore, all cases were treated surgically. Although endocrinological and pathological findings were available in most cases, the description of the clinical course was insufficient, making it difficult to analyze the treatment outcome. The need for a second surgery has been reported in several cases with Cushing’s disease or pituitary macro- (invasive) tumors [17,20,24,28,33,36,38,42,43,48,50], suggesting that these conditions may be risk factors for treatment resistance. It should be noted that a significant number of cases diagnosed with Cushing’s disease fail to achieve postoperative remission, leading to a second surgery [17,20,24,33,38,42,43]. This is especially true in cases of macro-tumors combined with ACTH-producing micro-tumors, which are difficult to detect on preoperative imaging [24]. In some cases, the GH and IGF-1 levels did not improve after the initial surgery, requiring re-operation for the GH-producing micro-tumors [48,50]. As acromegaly and Cushing’s disease are relatively common phenotypes of double PitNETs, the presence of hidden tumors should be considered during surgery for these conditions.

Recently, a clinical classification system for pituitary tumors has been proposed to guide treatment and prognosis, which scores several risk factors (e.g., tumor size, mass effect, invasion, residual tumor, and histopathology) and evaluates disease activity [78]. Because PitNETs include a variety of disease types, it is difficult to establish the appropriate indicators of disease status and prognosis, but such efforts will be useful in clinical practice. In double PitNETs, Zhang et al. analyzed the prognostic factors in 59 cases reported from 1990 to 2023 and found that the female gender and Cushing’s disease with a single lesion detected by surgery were associated with a poor prognosis, whereas double lesions detected by surgery and contiguous tumors were associated with a good prognosis [52]. However, it is difficult to conclude whether these are real prognostic factors because the number of cases analyzed was insufficient for the wide variety of double PitNETs. Although double PitNETs is infrequent, a large number of cases are needed to determine significant prognostic factors, and the clinical course of properly diagnosed cases is expected to increase.

## 11. Conclusions

The incidence of double PitNETs is very low, occurring in 0.1 to 1.2% of surgically resected PitNETs, and an accurate clinical diagnosis by endocrine examination is essential. It is often identified based on symptoms of hormone excess due to one of the two pituitary tumors, but our present analyses indicate that the proportion of endocrine-inactive cases and gonadotroph tumors are increasing recently. If two tumor-like lesions are seen on pituitary images, double PitNETs can be assumed preoperatively, but they are often detected as a single tumor. Multiple hormone excess with double lesions on MRI cannot be readily diagnosed. A definitive diagnosis requires pathology, including immunohistochemical analysis for pituitary hormones and transcription factors. Surgery is the mainstay of treatment, but attention should be paid to hidden hormone-producing micro-tumors. Double PitNETs are of great interest to endocrinologists, pituitary neurosurgeons, and pathologists because of the wide variety of clinical manifestations, radiological features, and pathological findings. Diagnostic and therapeutic strategies have improved, along with the advanced medical technologies for PitNET.

## Figures and Tables

**Table 1:** Clinical features in double PitNETs.

Clinical Feature	Number (% of All Cases)	
Any feature (*)		
Acromegaly	61	(45.5)
Cushing	47	(35.1)
Prolactinoma	24	(17.9)
TSHoma	2	(1.5)
Gonadotropinoma	1	(0.0)
None	17	(12.7)
Double features		
Acromegaly + Cushing	3	(2.2)
Acromegaly + Prolactinoma	6	(4.5)
Cushing + Prolactinoma	7	(5.2)

* Several overlapping cases exist.

**Table 2:** Combinations of IHC results in double PitNETs.

First PitNET	Second PitNET						
	GH	ACTH	PRL	LH/FSH	TSH	Others	Total
GH	22	22	7	22	2	7	82
ACTH	22	10	27	11	0	6	76
PRL	7	27	4	8	1	3	50
LH/FSH	22	11	8	4	0	2	47
TSH	2	0	1	0	0	0	3
Others	7	6	3	2	0	8	26
Total	82	76	50	47	3	26	284

**Table 3:** Chronological comparison of IHC results in double PitNETs.

IHC	Results	1980–2009	2010–2024	Total	*p* Value
GH	(+) (−)	35 81	47 121	82 202	0.69
ACTH	(+) (−)	32 84	44 124	76 208	0.79
PRL	(+) (−)	27 89	23 145	50 234	0.04 *
LH/FSH	(+) (−)	8 108	39 129	47 237	<0.01 *

* Statistically significant values from 1980 to 2009 and from 2010 to 2024.

**Table 4:** Radiological findings in double PitNETs.

Pituitary MRI Findings	Number (% of All Cases)	
Single tumor		
Macro-tumor	26	(31.7)
Micro-tumor	13	(15.9)
Double tumors		
Two macro-tumors	8	(9.8)
Two micro-tumors	12	(14.6)
Macro-tumor + Micro-tumor	17	(20.7)
No tumor		
None	6	(7.3)

## Abbreviations

The following abbreviations are used in this manuscript:

ACTH	adrenocorticotropic hormone
FSH	follicle-stimulating hormone
FT3	free triiodothyronine
FT4	free thyroxine
GH	growth hormone
IGF-1	insulin-like growth factor 1
IHC	immunohistochemistry
LH	luteinizing hormone
MRI	magnetic resonance imaging
PIT1	pituitary transcription factor 1
PitNET	pituitary neuroendocrine tumor
PRL	prolactin
SF1	steroidogenic factor 1
TPIT	T-Box family member TRX19
TSH	thyroid-stimulating hormone
TSHoma	thyroid-stimulating hormone producing tumor
